# Mobility and Navigation among the Yucatec Maya: Sex Differences Reflect Parental Investment, Not Mating Competition

**DOI:** 10.1007/s12110-015-9250-7

**Published:** 2015-12-09

**Authors:** Elizabeth Cashdan, Karen L. Kramer, Helen E. Davis, Lace Padilla, Russell D. Greaves

**Affiliations:** Department of Anthropology, University of Utah, Salt Lake City, UT USA; Department of Psychology, University of Utah, Salt Lake City, UT USA; Peabody Museum of Archaeology and Ethnology, Harvard University, Cambridge, MA USA

**Keywords:** Navigation, Range size, Mobility, Monogamy, Parenting, Sex differences, Maya

## Abstract

Sex differences in range size and navigation are widely reported, with males traveling farther than females, being less spatially anxious, and in many studies navigating more effectively. One explanation holds that these differences are the result of sexual selection, with larger ranges conferring mating benefits on males, while another explanation focuses on greater parenting costs that large ranges impose on reproductive-aged females. We evaluated these arguments with data from a community of highly monogamous Maya farmers. Maya men and women do not differ in distance traveled over the region during the mate-seeking years, suggesting that mating competition does not affect range size in this monogamous population. However, men’s regional and daily travel increases after marriage, apparently in pursuit of resources that benefit families, whereas women reduce their daily travel after marriage. This suggests that parental effort is more important than mating effort in this population. Despite the relatively modest overall sex difference in mobility, Maya men were less spatially anxious than women, thought themselves to be better navigators, and pointed more accurately to distant locations. A structural equation model showed that the sex by marital status interaction had a direct effect on mobility, with a weaker indirect effect of sex on mobility mediated by navigational ability.

Humans are often described as a mildly polygynous species, an apt characterization based on cross-cultural patterns of marriage and mating, and on sexual dimorphisms in body, behavior, and mating psychology (Geary 2010; Low 2015; Plavcan 2000; Kramer and Russell 2015). Sex differences in mobility and spatial ability also are widespread, and it has been suggested that they, like other sexually dimorphic traits, may have been shaped by sexual selection (e.g., Gaulin 1992; Jones et al. 2003). Because large ranges pose greater navigational and spatial challenges, it is plausible that these sex differences are related. Our aim in this paper is to consider two evolutionary arguments that have been proposed to explain these sex differences, with a focus on mating benefits and parenting costs.

Males show an advantage in many geometric spatial abilities (Halpern 2013; Kimura 2000; Voyer et al. 1995), and some recent studies have shown a sex difference in mental rotation as early as infancy (Moore and Johnson 2011; Quinn and Liben 2014). Navigational tasks show a less consistent male advantage, but where a sex difference exists it also tends to favor males (Coluccia and Louse 2004). Males also are more likely to use geometric and global reference cues when navigating, which facilitates long-distance travel in novel areas (Chai and Jacobs 2009; Lawton and Kallai 2002; Sandstrom et al. 1998; Ward et al. 1986). Beginning in middle childhood, boys travel farther than girls cross-culturally (Hart 1979; Matthews 1987; Whiting and Edwards 1992), and a similar pattern is widespread (although not universal) among adults in a variety of contexts and scales of distance. Greater male travel distances have been described for hunter-gatherer foraging ranges (Greaves 2006; Hurtado et al. 1992; Marlowe 2010; Tanno 1976), mating and exploration ranges (MacDonald and Hewlett 1999; Vashro and Cashdan 2015), and customary weekly travel among urban residents (Ecuyer-Dab and Robert 2004a). Although there is considerable cross-cultural variation arising from local environmental, economic, and social factors, the broad patterning in these sex differences has prompted researchers to look for evolutionary explanations.

Cross-species patterns lend support to sexual selection arguments for these sex differences. A male advantage in spatial ability is associated with a larger range size in several polygynous species where males gain a reproductive advantage from patrolling multiple female territories (Carazo et al. 2014; Gaulin and Fitzgerald 1986, 1989; Jasarevica et al. 2012; Perdue et al. 2011). The importance of mating competition is underscored by the finding that dimorphism in range size and spatial ability is found in polygynous vole species, but not monogamous ones (Gaulin and FitzGerald 1986, 1989). Humans do not find mates the way voles do, but two recent studies in small-scale societies suggest that mating competition may underlie sexually dimorphic range size and spatial ability in humans as well. Among Twe forager-farmers in Namibia, spatial ability was associated with yearly visiting range size, and men with larger ranges had more mates (Vashro and Cashdan 2015). Among Tsimané farmers in Bolivia, men traveled farther than women only during the mate-seeking years of late adolescence and early adulthood (Miner et al. 2014).

Yet humans are an extraordinarily variable species whose mating patterns vary from extreme monogamy to extreme polygyny, and most human behavior is highly facultative. We might therefore predict that behaviors related to mating competition, perhaps including range size and spatial ability, would vary with the degree of male mating competition and would be reduced in more monogamous populations. In other words, humans should exhibit a norm of reaction in range size in response to variation in mating competition and polygyny. Less sexually dimorphic range sizes may in turn lead to less sexually dimorphic spatial ability since spatial ability also is shaped by environmental and spatial experience (Baenninger and Newcombe 1989; Uttal et al. 2013). We evaluate these expectations in our study of a highly monogamous traditional farming population, the Maya. In contrast to the Tsimané and especially to the Twe, mating competition is minimal among the Maya: everyone marries, but only once, and extra-pair matings appear to be rare.

Another hypothesis for the sex differences in range size and spatial ability lies in the likelihood that travel imposes greater risks and fitness costs on reproductive-aged women than on men, owing to their greater investment in offspring (Sherry and Hampson 1997). Campbell (1999) has argued that this has made women more harm-avoidant generally, and more averse to taking physical risks (Byrnes et al. 1999). Females also are more spatially anxious than males (Lawton 1994; Lawton and Kallai 2002; Schmitz 1997), which may reflect these costs and may mediate their smaller ranges. Selection pressures from male mating competition and female harm-avoidance may both be important in shaping the sex differences in range size and spatial ability (Ecuyer-Dab and Robert 2004b).

Finally, subsistence patterns also have a major influence on mobility patterns, and cross-cultural regularities in the sexual division of labor often lead to greater male mobility, particularly with respect to the navigational and task demands of hunting and gathering (e.g., Eals and Silverman 1994; Greaves 2006; Hilton and Greaves 2008; New et al. 2007; Ogilvie 2006). For example, hunting, particularly pursuit hunting, is more likely than gathering to require travel over large areas and novel terrain, which would place greater navigational demands on men. Conversely, childcare responsibilities constrain the type of tasks that women typically do (Brown 1970; Hurtado et al. 1985; Kramer 2009). Although particular task demands vary widely, the mating and parenting concerns discussed above play a large role in shaping the broader patterns of the sexual division of labor.

We will evaluate these arguments using data from the Maya, with a focus on mating competition and parenting constraints. We think it likely that both have been important in shaping the sex difference in human mobility, although the effects of the former are likely to be more variable across cultures owing to the large variation in human mating patterns. This speaks strongly to the Maya case: if the mating hypothesis is as facultative as we expect, it would predict minimal sex differences in mobility in the mating years in this highly monogamous population. We expect that parenting constraints on women, in contrast, would be less variable across cultures, perhaps leading to a less facultative sex difference. If so, we would expect to see associated traits, such as spatial anxiety, found among the Maya and widely across cultures.

Our first aim is to assess whether there is a sex difference in mobility among the Maya and whether it is consistent with the facultative mating argument discussed above. As noted previously, mating competition is minimal among the Maya: marriage partners are found within a close-knit community, out-of-wedlock births and divorce do not occur, and remarriage is virtually unknown. We therefore expect that the sex difference in range size will be comparatively small in this population during the mate-seeking years. A sex difference in mobility resulting from parenting constraints on women, by contrast, would be most evident at older ages when people are caring for young children.

Our second aim is to construct an analytic model to determine how sex, navigational ability, spatial anxiety, and mobility are related in this population. If women are less mobile than men because of parenting constraints, it is plausible that greater spatial anxiety, reflective of greater parenting costs, would mediate that relationship. Navigational ability might also be expected to play a mediating role, although causation between range size and navigational skill could go both ways: males might have better navigational skill as an evolved consequence of selection pressures resulting from larger ranges, while learning from environmental experience is also likely to improve a person’s navigational proficiency.

## The Yucatec Maya

To test these expectations, data on spatial ability, spatial anxiety, and mobility were collected in a remote, rural Maya community in the interior of the Yucatan Peninsula, Mexico. Longitudinal economic, demographic, subsistence, and social trends have been studied in this community since the early 1990s (Kramer 2005; Kramer and Boone 2002; Kramer and McMillan 2006; Lee and Kramer 2002). Until recently, the community had limited access to wage labor, education, medical care, market opportunities, and goods. Although rapid economic development began to occur in the early 2000s when a paved road was built, most households today still subsist on small-scale maize cultivation, growing most of the food they consume, and selling small quantities of corn and honey to purchase basic household goods. Here we describe the population at large and focus on those factors that most directly affect age and sex differences in spatial ability, spatial anxiety, and mobility.

### Factors Affecting Sex Differences in Mobility and Range Size

Maya boys and girls are given great latitude from an early age to explore their environment independently. As soon as children are able to walk they are free to roam and frequently are found away from their family compounds, visiting friends and relatives or running errands. Much of a child’s day is spent outside in an unstructured landscape where they participate in unsupervised play and subsistence activities (field work, collecting firewood and water) and often are unaccompanied by adults. Even though today children spend more time at school, how children grow up in terms of spatial exploration has changed little and is similar across the age range of study participants.

Young adults are more likely to be educated and engage in wage labor than older adults. Between 1993 and 2013 completed education among 18–25-year-olds increased from a mean of 4.5 years to 9.4 years. Today, most children attend the local school until about the age of 13, with no sex difference in completed years of schooling (males *M* = 9.6, *SD* = 2.9; females *M* = 9.3, *SD* = 2.7; *t*_91_ = 0.53, *p* = 0.60). During their teens, opportunities to leave the village increase both for education and wage labor. At this time individual differences, but not sex differences, begin to emerge in how and where people spend their time. Since 2002 and the complete devastation of crops by hurricane Isidora, unmarried adults more commonly work outside the community for several years before marrying, at which time all women and most men quit their wage jobs and return to the village. In 2014, equal numbers of males (*n* = 12; 10%) and females (*n* = 11; 13%) ages 18–30 worked outside the community. All female wage laborers are unmarried, whereas several male wage workers are married. Wage work can take both males and females some distance from home, although they maintain close ties and often return on the weekends.

In contrast to young (single) adults, married men and women differ in how and where they allocate their time, with women typically traveling with others and men often traveling alone. Married men spend much of their day traveling to and from agricultural fields or into the forest to hunt and collect thatch or other forest products. Recently, farmers have expanded into remote and previously uncultivated areas of their territory, and into even more remote areas to establish apiaries. Consequently, men often are alone for long hours in the forest. Although married women’s spatial lives are more focused in the village, women travel in small parties into the forest to collect firewood, medicinal herbs and forest fruits, or they accompany other family members to work in the fields. Nursing women spend less time in the field than non-nursing women, and mothers of nursing infants less than a year old do no field work (Kramer 2009).

Navigation can be challenging in this environment because the densely vegetated forest conceals landmarks, and the sun often is obscured by the vegetation and cloud cover. The Maya do not have cell phones or GPSs and trails often are ephemeral. During interviews, older Maya men were forthcoming in talking about their discomfort at becoming lost, and most had been lost in the forest at some point in their adult lives. Younger men were commonly unconcerned about losing their way, but in many cases had never been lost. Women also report having been lost, but they rarely travel alone and seldom are at great distances from the village. Men, women, and children are well acquainted with other Maya communities, and from a young age they travel away from home to visit family, market towns or health clinics, or attend fiestas and religious functions.

In sum, although mobility for males and females at all ages has generally increased in recent years, study participants grew up under similar conditions in terms of having great freedom to explore their environment from an early age. Young adults, especially females, travel more than previously and are equally engaged in wage labor and education. However, following marriage, sex differences in mobility become pronounced.

### Monogamous Marriage and Low Variance in Male Quality

Marriages are stable and monogamous for life. No married adult (*n* = 214) living in the village has been divorced, and divorce has never been documented in the reproductive histories collected annually over the past 22 years. Widows and widowers are usually well into their fifties or sixties when they lose their spouse and do not remarry, with only one known exception. Of adults 30 and older, 86% of women (*n* = 80) and 95% (*n* = 77) of men are married or widowed. Although interactions with outsiders have increased, most marriage partners (92%) are from within the community, and rates of exogamous marriage are similar for males (9.7%, *n* = 11) and females (10.4%, *n* = 11).

Maya girls are not strongly sheltered or guarded. However, in the past they had little opportunity to pursue relationships on their own. Although travel to boarding schools and wage labor jobs likely has increased exposure to premarital conception, out-of-wedlock births are unknown. If a young woman conceives while single, she quickly marries. The Maya are forthcoming and open about their relationships, and although paternity is difficult to assess, there is little evidence of extramarital affairs, and only one case is known out of 150 reproductive histories collected in the past 20 years.

Husbands and wives share the same house, jointly discuss many household decisions and enjoy affable marriages and family relations. Because of the prevalence of lifelong monogamy, male and female fertility rates are nearly identical. Of married adults 40 and older, females have a mean completed fertility of 6.0 children (*SD* = 2.8, *n* = 54) and men, 5.91 children (*SD* = 2.6, *n* = 58). Compared with comparable small-scale societies, variance in male fertility is very low (Betzig 2012).

The adherence to monogamy is perhaps best understood in the context of several historic factors that contribute to low variance in male quality, and a lack of competition for mates or access to resources. The *ejido* land tenure system, instituted following the Mexican revolution, allocated to each community collectively owned land for growing crops and hunting, pasturage, and woodland. Ejido lands cannot be bought, sold, or inherited by individuals. This immutability, combined with constraints on transporting crops to market, greatly limits competition for arable land or the accumulation of property or wealth from surplus crop production. These factors minimize socioeconomic variance and the ability of individuals to monopolize resources, and they level opportunities for some males to be more successful in mate competition. Although the potential for stratification is emerging for the first time (variance in the amount of land under cultivation has significantly increased; *F* = 8.04, *p* = 0.005, Levene’s test), differences in male quality are not yet evident. This may be because in a rapidly changing and uncertain economic and social environment, status is difficult to establish. For example, the market price of maize almost halved in 2014, leaving those males who had invested in mechanized farming in large amounts of debt.

## Methods

To evaluate sex differences in navigational ability, spatial anxiety, and mobility, 148 Maya adults ages 15–50 participated in the 2014 study. Interviews and testing were conducted by Greaves and Kramer in Spanish or Maya, depending on the Spanish fluency of the participant. Half of the village’s adult population contributed to the study (Table 1), and testing took place in the participant’s household compound. The village is closely nucleated, and all compounds are situated within a kilometer of each other.

**Table 1:** Sample characteristics of Maya spatial study participants

	Male (*n* = 70)	Female (*n* = 78)
Average age	26.4 (*SD* = 11.4)	29.5 (*SD* = 11.7)
% married	44%	65%
Average age at first birth	22.5 (*SD* = 3.2)	21.8 (*SD* = 4.5)
Average number of children	4.0 (*SD* = 2.5)	3.4 (*SD* = 4.5)

### Mobility

Participants were asked about 20 locations in the region, and for each place they were asked whether they had been there never, once, a few times, or many times. This forms a 4-point ordinal scale (0–3) for each location. The average over the 20 locations is our mobility measure for each individual. This measure incorporates both number of places visited and frequency with which they have been visited. It is a cumulative measure of how far people have traveled over their lives, and it reflects mobility over a wide region rather than daily subsistence travel. Data on daily mobility from a 2010 time allocation study were also available for a subset of the sample.

### Spatial Anxiety

We measured spatial anxiety with four questions designed to assess how anxious or fearful people felt about navigating in novel areas. For example, one question was, “If you made a wrong turn when you were out alone and did not recognize where you were, would you be concerned that you might not find your way home?” Answers were scored on a 3-point ordinal scale (not anxious, somewhat anxious, anxious). The average over the four questions is our measure of spatial anxiety, with larger numbers indicating greater anxiety.

### Navigational Ability: Pointing Error

Average error in a pointing task was used as an indicator of navigational ability. Participants were asked to point to seven locations in the region using a Brunton Pocket Transit International Compass mounted on a tripod, with the compass sight extended to form the pointing indicator. Correct bearings were calculated from GPS coordinates, and the absolute value of the difference between the correct and the pointed bearing, in degrees, is our measure of pointing error. Of the seven locations, the two farthest were removed during analysis because of missing values and high pointing error. Men’s and women’s error distributions for these two locations were indistinguishable, suggesting unfamiliarity with these locations rather than a testable difference in pointing accuracy. The five remaining locations were at an average distance of 33.3 km (*SD* = 15.4 km, range 12.7–49.0 km) from the community.

### Navigational Ability: Wayfinding by Self-Report

We asked participants three questions to assess their wayfinding ability, each of which could be answered with a 3-point ordinal response. Participants were asked how readily they could point north when they were away from camp, whether they or others usually do the navigating when traveling with others, and whether or not they are good at giving directions. Higher numbers indicate better wayfinding ability. These questions were adapted from the longer Santa Barbara Sense of Direction Scale (Hegarty et al. 2002), which has been validated in Western populations with behavioral navigation measures.

Sex-difference analyses, multivariate structural equation modeling, and graphic productions were performed with R (version 3.1.2) and AMOS (version 22).

## Results

### Sex Differences

Our first goal was to evaluate whether the sex differences frequently reported for other societies are also found among the Maya.

Women reported significantly higher levels of spatial anxiety than men (*t*_150_ = −2.16, *p* = 0.03) and pointed less accurately (*t*_139_ = −3.6, *p* < 0.001), whereas men reported better sense of direction, *t*_149_ = 4.3, *p* < 0.001 (see Table 2 for means). To see whether age moderated these relationships, we added age and age-squared (since the effect of age is likely to be nonlinear) as predictors in separate regressions on the three dependent variables. Sex remained a significant predictor of spatial anxiety, pointing error, and sense of direction, whereas age had no effect.

**Table 2:** Descriptive statistics for the five spatial variables

	*Mean SD*		*n*		Cohen’s *d*
	Males	Females	Males	Females	
Spatial anxiety (Scale 0–4)	2.4 (0.4)	2.6 (0.4)	65	78	.50*
Pointing error (degrees)	28.0 (13.2)	37.0 (15.6)	70	67	.67**
Wayfinding (Scale 0–4)	2.12 (0.5)	1.78 (0.5)	75	78	.68**
Mobility	27.2 (7.9)	26.3 (7.8)	65	68	.13

Spatial anxiety and wayfinding are ordinal scales derived from interviews; pointing error is absolute degrees of error (out of 360) in pointing to known distant locations; mobility is an ordinal scale based on the number and frequency of visits to 20 locations in the region. Cohen’s *d* is the effect size used to indicate the standardized difference between the male and female means. **p < 0.001, *p < 0.05 (*t*-test)

**Fig. 1:**
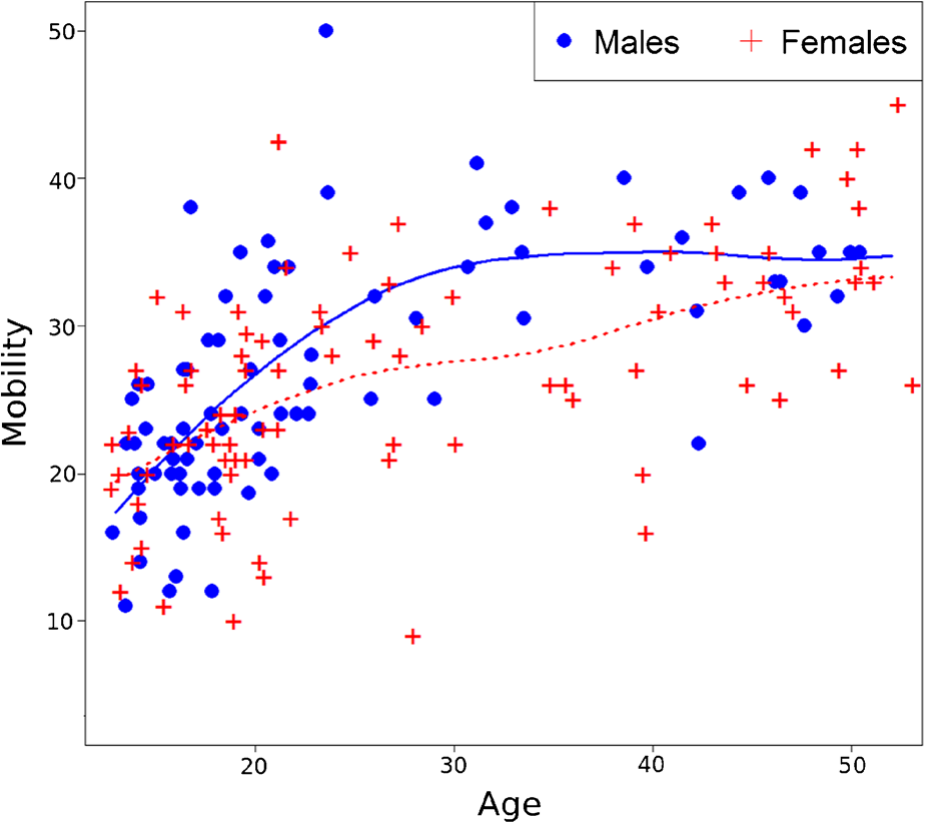
Scatterplot of age by mobility stratified by sex, with loess curves for males (*solid line*) and females (*dotted line*). Mobility is an ordinal scale based on the number and frequency of visits to 20 locations within the region

**Fig. 2:**
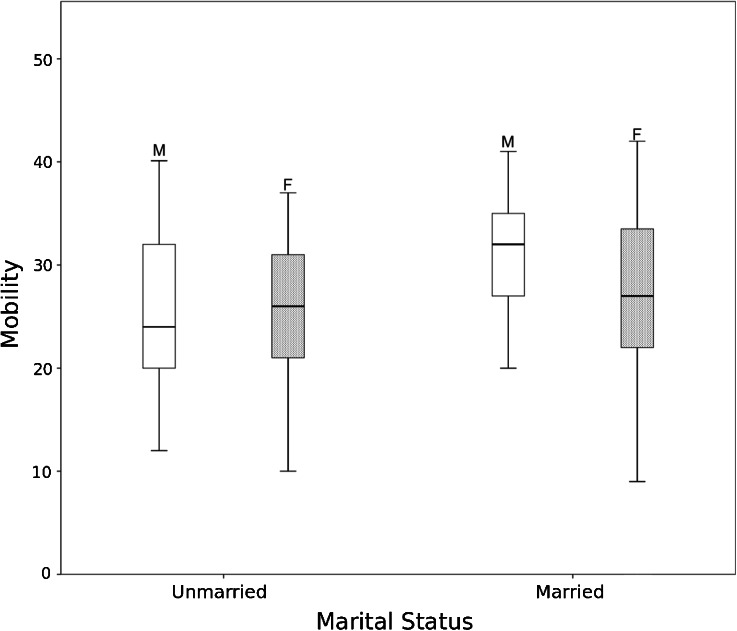
Boxplots of mobility for married and unmarried Maya males and females between 15 and 50 (*n* = 148). Mobility is an ordinal scale based on the number and frequency of visits to 20 locations in the region. Mobility, controlling for age, is significantly greater for married men compared to unmarried men, *t*_73_ = −3.67, *p* < 0.001). Difference between married and unmarried women, and unmarried women and unmarried men, is not significant

Mobility was not significantly different when the entire sample of men and women was considered across the lifespan (*t*_154_ = 0.70, *p* = 0.48). This is the case even when we control for age and age-squared. However, marriage had a strong effect on men’s mobility. Separate analyses for men and women show that when both age and marital status are entered into the model, mobility is significantly greater among married men (*M* = 1.17, *SD* = 0.45) than unmarried men (*M* = 0.67, *SD* = 0.07; *t*_73_ = −3.67, *p* < 0.001), whereas marital status had no effect on the mobility of women (*t*_79_ = −0.51, *p* = 0.61) (Figs. 1 and 2). Unmarried Maya men and women did not differ in their mobility (*t*_95_ = 0.05, *p* = 0.96), but the sex difference becomes significant after marriage. Married men had significantly greater mobility than married women (*t*_62_ = −2.13, *p* = 0.04), which can account for the sex difference in mobility in middle age seen in Fig. 1.

### Relationship among Navigational Ability, Anxiety, and Mobility

We used structural equation modeling (SEM) to evaluate other possible indirect effects that could help explain variation in mobility. One implication of the parenting hypothesis is that the risks and costs of travel have selected for greater spatial anxiety in women, and that women in consequence do not travel as far as men. We therefore considered spatial anxiety as a possible mediator between sex and mobility. If larger ranges are a selection pressure favoring better navigational ability, then navigational ability is also a possible mediator between sex and mobility. We have two variables that measure navigational performance: pointing error (a behavioral measure) and the wayfinding questions (a self-report measure). We used these two variables to construct the latent variable “navigational ability.” For the SEM analysis, we reversed the directionality of pointing error, calling it “pointing accuracy,” so that all the navigational measures are in the same direction (higher scores meaning better navigation). Because mobility increased after marriage with men but not with women, we included an interaction term of sex by marital status. Since mobility is a cumulative measure, we also controlled for age and used the residual of mobility by age in the model.^1^

**Fig. 3:**
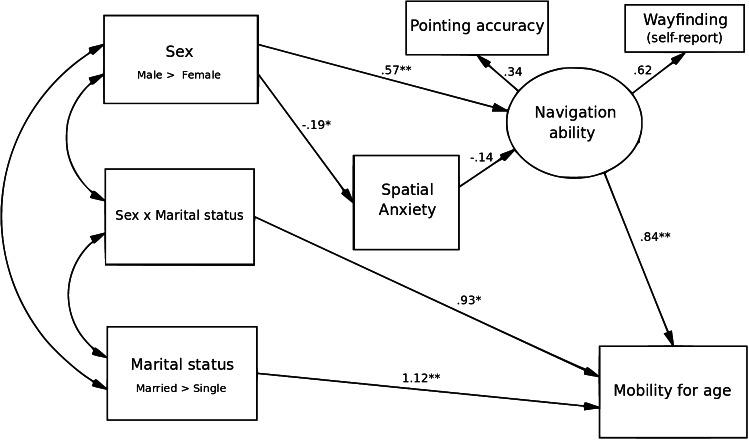
Results of SEM analysis, with standardized path coefficients. Navigation ability is a latent variable, constructed from the measured variables wayfinding and pointing accuracy. Larger numbers on the navigation variables indicate better navigation, and larger numbers for spatial anxiety indicates greater anxiety. Mobility for age is the residual of mobility by age. **p < 0.01, *p < 0.05

We initially ran a model that included all these variables as predictors of mobility. It included direct effects on mobility of marital status and of the sex-by-marital-status interaction, and indirect effects of sex on mobility, mediated by spatial anxiety and navigational performance. This model is similar to that shown in Fig. 3, but with a direct path from spatial anxiety to mobility. Multivariate analysis by structural equation modeling (SEM) was conducted using maximum likelihood estimation. To account for missing data points, full information maximum likelihood (IML) measures used all available data in order to generate maximum-likelihood-based statistics. Goodness-of-fit was assessed for the overall model, and although the fit measures approached acceptable levels, the effect of spatial anxiety on mobility was not significant. We therefore tried a second model, as shown in Fig. 3, with spatial anxiety affecting mobility through navigational ability. Standard goodness-of-fit measures for this model are acceptable (χ^2^ = 14.36, RMSEA 0.038, CFI 0.996). RMSEA (root mean square error of approximation) values less than 0.08 and CFI (comparative fit index) values more than 0.9 indicate that the model adequately represents the data (Browne and Cudeck 1993).

This model, shown in Fig. 3, indicates that marriage and the interaction effect of marriage by sex both had direct positive effects on mobility (β = 1.12, *p* = 0.003 and β = 0.93, *p* = 0.017, respectively), supporting earlier analyses showing that married individuals were more mobile and that married men had greater mobility than married women, unmarried men, and unmarried women. Sex had an indirect effect, with men having better navigational performance, and better navigational performance in turn predicting greater mobility. Women also reported greater spatial anxiety. The indirect effect of spatial anxiety on mobility was in the expected direction but did not reach statistical significance; however, its inclusion did make the model perform better overall.

### Marital Status and Sex

Given these results, we wanted to know why marriage had a positive effect on mobility for men. The result was not anticipated, and a similar effect was not found for women. We therefore categorized the 20 locations used to calculate mobility by the primary reason for visiting each location. Destinations were divided into four categories: locations to visit kin, locations to procure resources, wage labor locations, and other (hospital or religious trips). Married men were significantly more likely than unmarried men to visit locations to procure resources (*t*_78_ = −3*.*21, *p* = 0.002) and to engage in wage labor (*t*_66_ = −2*.*92, *p* = 0.005).

**Fig. 4:**
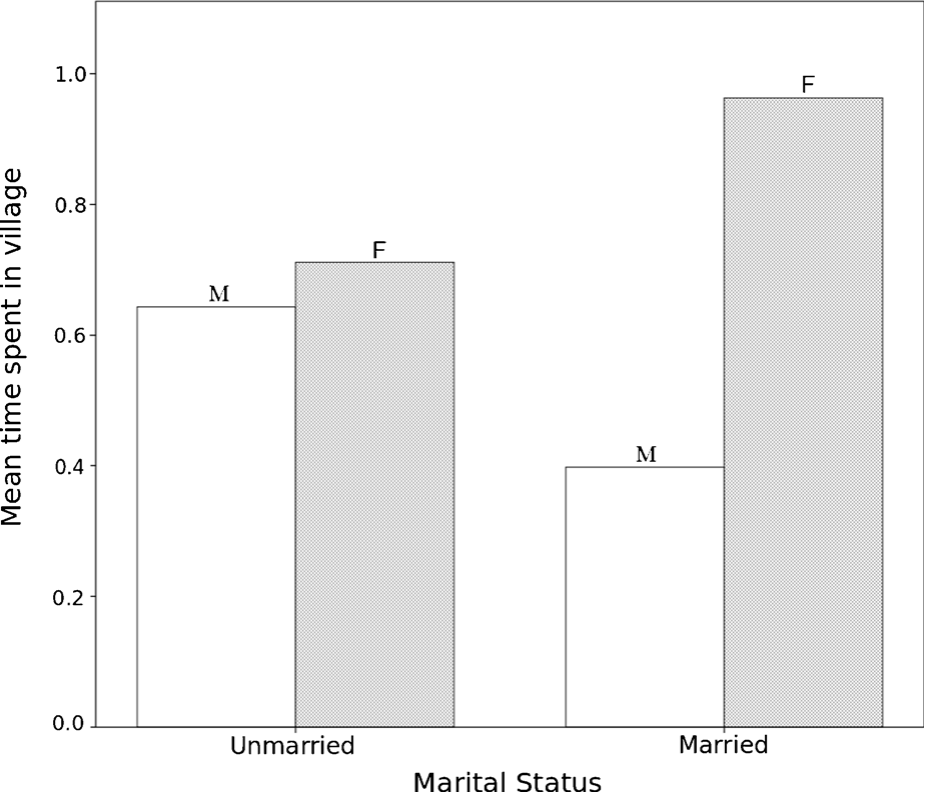
Proportion of time spent within the village for men and women before and after marriage. Data based on scan samples (*n* = 5419) of 15 males and 23 females, ages 15–50. Time not in the villages includes time spent in the fields and forest, as well as in other more distant communities

In order to see whether daily travel showed the same effect as regional mobility, we analyzed time allocation data collected in 2010. The data are for a subset of the sample so could not be included in the path model. Scan samples (*n* = 5419) were collected on male (*n* = 15) and female (*n* = 23) adults ages 15–50, and daylight observations were aggregated by location to indicate the proportion of time an individual spent in or out of the village (Fig. 4). Time out of the village includes time spent in the fields and forest, as well as in other more distant communities. Before marriage, there was no difference between the proportion of time spent in the village by men (*M* = 0.66, *SD* = 0.28) and women (*M* = 0.67, *SD* = 0.31, *t*_11_ = −0.05, *p* = 0.96). After marriage, however, the proportion of time spent in the village decreases for men (*M* = 0.39, *SD* = 0.16) and increases for women (*M* = 0.92, *SD* = 0.17) such that married men spend more than twice as much time as married women out of the village (*t*_21_ = −7.66, *p* < 0.001; Fig. 4). The change with marital status is significant for both women (*t*_44_ = 3.40, *p* = 0.002) and men (*t*_28_ = 3.24, *p* = 0.003).

## Discussion

### Sex Differences in Spatial Ability and Range Size

Maya men on average performed better than women at our navigational tasks. They pointed more accurately to known but distant locations and had a better self-reported sense of direction. Women also reported more anxiety about getting lost and finding their way in unfamiliar locations.

It would be reasonable to expect these indicators of navigational ability and confidence to reflect larger male range sizes, but the result here is not straightforward. Men and women overall did not differ significantly in our primary measure of mobility, which was designed to capture mobility over the wider region. However, men do travel significantly farther after marriage whereas women do not. Path model results show that this interaction between sex and marital status had a direct effect on mobility. A smaller effect of sex on mobility was indirect, mediated by men’s better navigational ability.

The sex differences in navigational performance and spatial anxiety despite similar mobility is interesting. One interpretation could be simply that these sex differences are not locally calibrated to differential ranging experience. This could be the case with respect to spatial anxiety; a less facultative response would make sense to the extent that spatial anxiety reflects women’s greater harm-avoidance generally, in response to women’s universally greater parental investment (Campbell 1999).

The sex difference we found in navigation performance despite similar mobility is more likely to reflect limitations in our mobility measures. Larger ranges, other things being equal, pose more navigational challenges, but other factors are probably also at play in shaping sex differences in navigational experience, including the ways in which women and men travel. Unlike Maya men, Maya women do not drive or ride bicycles, and when they use motorized transport they do so as passengers, traveling with others. Because their experience as active navigators is limited to distances traveled on foot, they have less regional navigational experience despite their having traveled (as passengers) widely over the region, visiting many of the same places as men. They are also more likely to travel with others, even when traveling on foot. A similar argument was made to explain the sex difference in some spatial measures among Hadza foragers. As is common among foragers, Hadza women forage in groups whereas men hunt alone, which means that men are more dependent on their own sense of direction (Cashdan et al. 2012). A similar pattern is described for the Twe (Vashro et al. 2015). These factors may have played a role in shaping sex differences in navigational ability, over and above sex differences in range size.

### Reasons for Sex Differences in Range Size

One hypothesis for the sex difference in range size, particularly in “visiting” or “exploration” ranges (i.e., to other camps, villages or towns), is that men gain fitness benefits through increased mating opportunities. This scale of mobility has not received much attention from anthropologists, but two recent studies of polygynous forager-farmer groups, the Twe and Tsimané, found evidence linking larger male visiting ranges to mating competition, in support of this hypothesis (Miner et al. 2014; Vashro and Cashdan 2015).

Our mobility measure was also designed to capture mobility on this scale. However, we anticipated that the sex differences in range size would be low and not associated with mating competition in this population because the Maya are highly monogamous, as reflected both in their marital behavior (see ethnographic background section) and in their fertility. Variance in reproductive success (RS_var_) among Maya males is lower than in other small-scale populations, including both foragers and farmers (Betzig 2012), and is about the same for men (6.8) and women (7.8). This makes the Maya an interesting contrast to the more polygynous Twe (male RS_var_ = 19.7; Vashro, personal communication) and Tsimané (male RS_var_ = 19.9; von Rueden et al. 2011), where male mating competition appears to be one factor driving larger male visiting ranges. Although unusual for farming populations, the low Maya RS_var_ is consistent with the low variance in male socioeconomic success described earlier, and with the apparent absence of behaviors associated with male mating competition. It is also consistent with our finding that there is no sex difference in range size among unmarried Maya adults.

We expected that any sex difference in regional range size among the Maya would be associated with parenting costs rather than mating competition, and that it would result from reduced mobility among women owing to the greater fitness costs they incur from travel. In addition to the direct constraints of childcare on mobility, this expectation is due to the fact that the risks of being hurt or lost while traveling exact a larger fitness cost on women since young children are less likely to survive a mother’s death or injury than a father’s. This would be a special case of the general evolutionary argument for women’s lower fear threshold and aversion to taking physical risks (Campbell 1999).

The mobility data, however, indicate that on a regional level, married men are traveling more, not that married women are traveling less. Marriage and parenting responsibilities are closely linked among the Maya since they neither divorce nor have children out of wedlock. A post-hoc analysis of travel destinations indicates that married men increase travel to market towns, where they can sell and exchange agricultural products, and to administrative towns, which are centers for wage work. Although we cannot know for certain, our inference is that because mobility changes with marital status, married men increase their range size and seek these destinations for work and subsistence reasons, which provides support for their families. More direct evidence about the motive for long-distance travel is clearly needed.

Because the mobility measure is cumulative, it is limited in terms of what it can tell us about current mobility constraints and motives. It also measures only long-distance travel to other villages and towns, not daily or local travel to fields and forest. In order to capture sex differences in daily mobility, we used scan sample data collected for a subset of this sample in 2010. As with our primary mobility measure, unmarried men and women do not differ in the proportion of time they spend away from the village. After marriage, however, married men are spending significantly more time out of the village, and married women significantly less.

Taken together, these data support the argument that in this highly monogamous population, parenting, rather than mating competition, leads to a modest sex difference in mobility during the childbearing years, and that it results from both constraints on mobility by married women and subsistence-related travel by married men. This result is consistent with the fact that male and female reproductive interests are closely aligned in this monogamous population, and it serves as a useful counterpoint to the regional mobility patterns described for more polygynous societies.

## Footnotes

1 Weighted effect codes were applied to the variables “sex” and “marital status” in the SEM (Cohen et al. 2003). With weighted effects coding, males were coded as 1 and females as −0.93, where 0.93 is the ratio of females to males in the sample. Similarly, married individuals were coded as 1 and unmarried persons as −0.86, where 0.86 is the ratio of unmarried to married individuals in the sample.
